# Association of *BMAL1* and *CLOCK* Gene Polymorphisms with Preeclampsia Risk with Subtype Analysis

**DOI:** 10.3390/ijms262110797

**Published:** 2025-11-06

**Authors:** Fan Xia, Peiwen Wang, Ziye Li, Jiehua Wei, Jianhui Wei, Yuhang Wu, Chu Liu, Shanyu Lin, Suyan Guo, Linbin He, Mengshi Chen, Lizhang Chen, Tingting Wang

**Affiliations:** 1 Department of Epidemiology and Health Statistics, Xiangya School of Public Health, Central South University, No. 172 Tongzipo Road, Yuelu District, Changsha 410013, China; 226901005@csu.edu.cn (F.X.); 31220149@csu.edu.cn (P.W.); liziye_ah@163.com (Z.L.); weijh2021@163.com (J.W.); weijihi@163.com (J.W.); 226901015@csu.edu.cn (Y.W.); 210201014@csu.edu.cn (C.L.); shanyu0812@126.com (S.L.); guosuyan0815@163.com (S.G.); helinbin2023@163.com (L.H.); mancechen@foxmail.com (M.C.); 2 Hunan Provincial Key Laboratory of Clinical Epidemiology, Xiangya School of Public Health, Central South University, No. 172 Tongzipo Road, Yuelu District, Changsha 410013, China

**Keywords:** preeclampsia, circadian rhythm, pregnancy, *BMAL1*, *CLOCK*

## Abstract

Preeclampsia (PE), a major cause of maternal and perinatal morbidity, is a hypertensive pregnancy disorder with poorly defined pathogenesis. While dysregulation of core circadian genes including brain and muscle ARNT-like 1 (*BMAL1*; also termed *ARNTL*) and circadian locomotor output cycles kaput (*CLOCK*) has been implicated in PE, the contribution of their genetic polymorphisms to PE remains unclear. In this case–control study, polymorphisms in *BMAL1* and *CLOCK* were genotyped using MassARRAY in 202 PE patients (97 early-onset [eoPE], 105 late-onset [loPE]) and 400 controls. Following genotyping and linkage disequilibrium-pruning (*r*^2^ > 0.8) to retain representative tag SNPs, the final set for association analysis comprised three non-redundant *BMAL1* SNPs (rs4757144, rs11022780, rs969485) and one *CLOCK* SNP (rs1048004). After confounder adjustment, no significant associations were detected for *CLOCK* variants, whereas the *BMAL1* rs11022780 variant demonstrated a significant protective effect against PE (TT vs. CC: OR = 0.26 [95% CI 0.09–0.78]; recessive model: OR = 0.25 [95% CI 0.09–0.74]), particularly in the eoPE subgroup. Expression quantitative trait locus (eQTL) analysis confirmed that this SNP correlated with *BMAL1* mRNA expression in whole blood, and protein–protein interaction analysis highlighted BMAL1′s central role in circadian networks, implying a genetically influenced regulatory mechanism of PE through *BMAL1* expression.

## 1. Introduction

Preeclampsia (PE), a multifactorial hypertensive disorder arising after 20 weeks of gestation, is characterized by new-onset hypertension with proteinuria or end-organ dysfunction [1]. It is clinically categorized into early-onset PE (eoPE, diagnosed <34 weeks of gestation) and late-onset PE (loPE, diagnosed ≥34 weeks of gestation), the former of which is linked to considerably worse clinical outcomes [2,3]. Affecting approximately 2% to 8% of pregnancies worldwide, and 4% to 5% in China, PE poses serious risks to both maternal and fetal health [4,5]. It is associated with acute maternal complications such as HELLP syndrome, and elevates the long-term maternal risks of metabolic diseases including cardiovascular disease, chronic kidney disease, and type 2 diabetes mellitus [6,7,8,9,10,11]. Additionally, PE adversely affects the fetus, increasing the risks of growth restriction, preterm birth, and placental abruption [12]. As a leading cause of maternal mortality, PE results in an estimated 76,000 maternal deaths and 500,000 fetal or neonatal deaths globally each year [13]. Despite advances in medical technology, termination of pregnancy remains the only intervention that definitively resolves the underlying pathological cause of PE, namely placental dysfunction [14]. It is imperative to advance etiological research for identifying high-risk populations based on pathogenic mechanisms, thereby facilitating early screening and intervention to prevent PE onset, improve pregnancy outcomes, and reduce societal healthcare burdens.

Although the precise mechanisms underlying PE remain incompletely elucidated, two predominant pathophysiological theories have been proposed: the placental two-stage model and the maternal cardiometabolic maladaptation theory [15,16,17]. According to the two-stage model, the initial stage involves defective placental development due to impaired trophoblast invasion and inadequate remodeling of the spiral arteries, leading to reduced placental perfusion, hypoxia, and oxidative stress [17,18,19]. It is widely recognized that these placental alterations provoke the release of anti-angiogenic factors, such as soluble fms-like tyrosine kinase-1 (sFlt-1), and pro-inflammatory mediators into the maternal circulation, initiating the second stage—systemic endothelial dysfunction, inflammatory activation, and the clinical manifestations of PE [20,21,22,23]. EoPE is closely aligned with this model, characterized by profound placental insufficiency, elevated sFlt-1 levels, decreased vascular endothelial growth factor (VEGF) and placental growth factor (PlGF), and frequent fetal growth restriction [24,25,26,27]. Alternatively, the cardiometabolic theory posits that pre-existing maternal cardiovascular dysfunction or metabolic abnormalities—such as chronic hypertension, obesity, or insulin resistance—impair the hemodynamic adaptations to pregnancy, ultimately leading to placental hypoperfusion and PE [28,29,30,31]. It is observed that loPE is often associated with this etiology, wherein maternal factors predominate over primary placental defects. It is also noted that the clinical presentation is frequently less severe with respect to angiogenic imbalance and placental pathology [32,33,34].

Emerging evidence suggests that disruption of placental circadian rhythms may contribute significantly to the pathogenesis of PE [35,36]. Circadian rhythms are endogenous oscillations with a period of approximately 24 h that regulate numerous biological processes through molecular clockworks [37,38]. In mammals, these rhythms are coordinated by the suprachiasmatic nucleus (SCN) of the hypothalamus, which serves as the central pacemaker that synchronizes peripheral oscillators across various tissues via the hierarchical regulation of clock gene expression [39,40]. The core molecular clock operates through interlocked transcriptional-translational feedback loops involving key genes such as brain and muscle Arnt-like protein 1 (*BMAL1*, also termed *ARNTL*), circadian locomotor output cycles kaput (*CLOCK*), cryptochromes (*CRYs*) and period homolog proteins (*PERs*) [41], all of which are expressed in animal and human placental tissue [42,43,44,45,46] and extravillous trophoblast cell lines [47]. Notably, among these placental clock genes, circadian rhythmicity is exclusively observed in the expression of *BMAL1* and *CLOCK* [35,46]. Importantly, dysregulation of the *BMAL1* and *CLOCK* genes has been closely linked to core pathological mechanisms of PE, including imbalances in vascular endothelial dysfunction [48,49,50,51], as well as mitochondrial impairment and oxidative stress [52,53,54,55]. These observations position *BMAL1* and *CLOCK* genes as potential central players in PE development. The *BMAL1* gene is located on chromosome 11p15.3, while the *CLOCK* gene is located on chromosome 4q12. Altered expression of the *CLOCK* gene has been observed in placental tissue from PE patients [56]. However, given the practical challenges in obtaining placental tissue prenatally, there is a critical need to identify non-invasive biomarkers. Intriguingly, *BMAL1* and *CLOCK* gene polymorphisms have been associated with hypertension in a study of healthcare workers [57], supporting their potential role in PE pathologies. Therefore, we hypothesize that specific single nucleotide polymorphisms (SNPs) detectable in peripheral blood, which regulate *BMAL1* and *CLOCK* expression, could serve as genetic proxies for placental gene function and be associated with PE risk.

In summary, this case–control study aimed to investigate the associations of *BMAL1* and *CLOCK* gene polymorphisms with PE risk. Following genotyping and quality control that included testing for Hardy–Weinberg equilibrium (HWE) as well as linkage disequilibrium (LD) pruning, we evaluated the associations of single SNPs, haplotypes, and gene–environment interactions with PE risk. Given the potential etiological differences between early-onset and late-onset PE, the distribution of PE-associated variants was further analyzed across these subtypes. Expression quantitative trait locus (eQTL) analysis was performed to evaluate the correlations between these PE-associated risk loci and *BMAL1/CLOCK* gene expression levels in whole blood. Furthermore, a protein–protein interaction (PPI) network analysis was employed to uncover functional pathways implicated by PE-associated genes.

## 2. Results

### 2.1. Baseline Characteristics of Participants

A total of 602 pregnant women were included in this study, comprising 202 women with PE and 400 controls. As shown in Table 1, the baseline characteristics analysis indicated that there were no significant differences between the two groups in terms of obstetric history (including gravidity, parity, and history of adverse pregnancy), or certain behaviors and exposures during pregnancy (such as smoking, alcohol consumption, and tea consumption) (all *p* > 0.05). Additionally, no significant differences were observed between the groups in terms of folic acid supplementation, history of autoimmune disease, or the incidence of fever, respiratory tract, gastrointestinal, and urinary tract infections during early pregnancy (all *p* > 0.05).

However, a significantly higher proportion of women in the PE group, compared to the control group, were aged ≥35 years (27.72% vs. 12.50%), resided in rural areas (15.84% vs. 4.25%), were pre-pregnantly overweight or obese (38.61% vs. 8.75%), had a history of pregnancy complications (18.81% vs. 10.25%), had a history of anemia during pregnancy (18.81% vs. 12.50%), consumed tea during pregnancy (17.82% vs. 9.50%), had poor sleep quality during pregnancy (30.69% vs. 12.75%), had a daily sleep duration of less than 7 h (25.74% vs. 8.50%), had periodontitis in early pregnancy (7.92% vs. 3.75%) and had reproductive tract infection in early pregnancy (10.89% vs. 4.75%) (all *p* < 0.05). In contrast, the prevalence of secondhand smoke exposure during pregnancy was significantly lower in the PE group (31.68%) than in the control group (43.50%) (*p* < 0.05).

### 2.2. Associations of Gene Polymorphisms in BMAL1 and CLOCK with PE Risk Under the Codominant Model

The associations between core circadian rhythm gene polymorphisms (*BMAL1* and *CLOCK*) and PE risk were evaluated under a codominant model, with the results summarized in Table 2. All SNPs in controls adhered to HWE (*p* > 0.05). Under the codominant model, no significant associations were observed between PE risk and the following SNPs: rs4757144 and rs969485 in the *BMAL1* gene, and rs1048004 in the *CLOCK* gene (all *p* > 0.05). In contrast, rs11022780 in the *BMAL1* gene was significantly associated with PE after adjustment for confounders (*p* < 0.05).

At the *BMAL1* rs11022780 locus, the CC genotype was the most prevalent in both the PE (51.49%) and control (52.50%) groups, followed by the CT genotype (46.04% vs. 39.75%). The TT genotype was less frequent in the PE group (2.47%) compared to the control group (7.75%). After adjustment for potential confounders such as maternal age, residence, pre-pregnancy body mass index (BMI), and history of pregnancy complications, women with the TT genotype had a significantly lower risk of developing PE compared to those with the CC genotype (aOR = 0.26, 95% CI: 0.09–0.78).

### 2.3. Associations of Gene Polymorphisms in BMAL1 and CLOCK with PE Risk Under the Recessive Model

As shown in Table 3, under the recessive model, none of the SNPs examined—rs4757144 and rs969485 in the *BMAL1* gene, and rs1048004 in the *CLOCK* gene—demonstrated a statistically significant association with PE risk (all *p* > 0.05), which was consistent with the findings under the codominant model.

Following adjustment for confounders using multivariable logistic regression, the *BMAL1* rs11022780 polymorphism remained significantly associated with PE under the recessive model (TT vs. CC + CT: aOR = 0.25, 95% CI: 0.09–0.74). The TT genotype of rs11022780 consistently exhibited protective effects against PE across both codominant and recessive models (all *p* < 0.05).

### 2.4. Haplotype-Based and Gene–Environment Interaction Analysis of the BMAL1 Gene for PE Risk

Haplotype analysis of the three non-redundant *BMAL1* SNPs (rs4757144, rs11022780, and rs969485) identified seven common haplotypes with frequencies greater than 1%. However, none of these haplotypes showed a statistically significant association with PE risk (all *p* > 0.05; Supplementary Table S1).

Furthermore, additive and multiplicative interaction models were employed to evaluate the interactions between the PE-associated *BMAL1* rs11022780 polymorphism and sleep quality as well as daily sleep duration during pregnancy on PE risk, and showed no statistically significant additive or multiplicative interactions (all *p* > 0.05; Supplementary Table S2). These analyses were adjusted for key covariates including age, residence, pre-pregnancy BMI, history of pregnancy complications and anemia, secondhand smoke exposure, tea consumption, periodontitis and reproductive tract infection (Supplementary Table S2).

### 2.5. Associations Between BMAL1 Polymorphism and PE Subtypes

In this study, which included 202 women with PE (97 eoPE and 105 loPE), we further examined the association of the *BMAL1* rs11022780 polymorphism with PE subtypes under codominant and recessive models, accounting for their distinct pathogenesis (Table 4). No significant association was identified for this SNP with loPE (*p* > 0.05). Post hoc power calculations (Supplementary Methods S1) revealed limited statistical power for the loPE subgroup analyses (power = 25.4% for recessive model and 29.3 % for codominant model; Supplementary Table S3). Therefore, the lack of a significant association in the loPE subgroup should be interpreted with caution, as a true association might have been missed.

Under the codominant model, the overall test for rs11022780 revealed a significant association with eoPE risk (*p* = 0.029). However, when comparing individual genotypes against the reference (TT vs. CC), none reached statistical significance—a finding likely attributable to limited statistical power for this specific comparison (power = 55.8 %, Supplementary Table S3), as reflected in wide confidence intervals, and indicative of a non-additive genetic effect. Notably, under the recessive model, the TT genotype of rs11022780 demonstrated a significantly reduced risk of eoPE (aOR = 0.13, 95% CI: 0.02–0.98, *p* = 0.048). The detection of this association with 77.5% power (Supplementary Table S3), despite the limited sample size, suggests that the observed protective effect was sizable enough to be detected, yet the borderline *p*-value necessitates confirmation in larger cohorts.

### 2.6. Effects of PE-Associated BMAL1 Polymorphism on Gene Expression in Whole Blood

To explore the potential functional mechanism of the PE-associated SNP, we performed a cis-eQTL analysis using data from the first phase of the eQTLGen consortium [58]. As shown in Table 5, the rs11022780 variant significantly altered *BMAL1* expression in whole blood. The C allele of rs11022780 was associated with reduced *BMAL1* expression (Z = −5.269, FDR < 0.001). Consistent with this finding, the protective TT genotype of rs11022780 does not carry this low-expression allele, implying that its protective effect against PE may stem from the absence of a deleterious regulatory variant that downregulates *BMAL1*.

### 2.7. Protein–Protein Interaction Network Analysis of BMAL1

**Figure 1:**
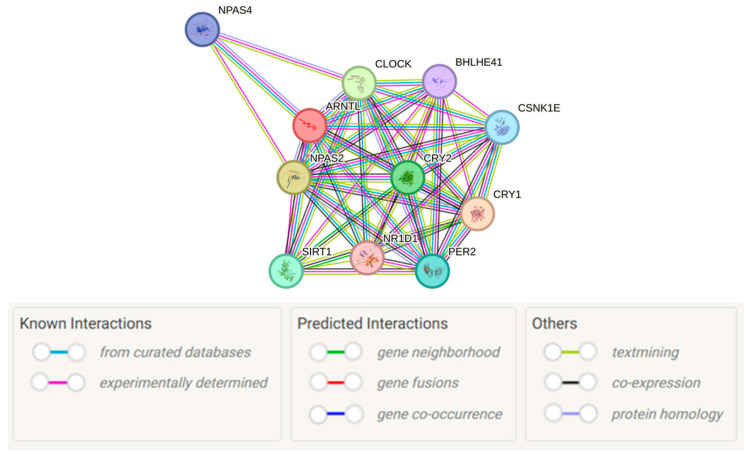
Protein–protein interaction network of BMAL1 (ARNTL). The network was generated using the STRING database (version 12.0) [59]. Image obtained from STRING (https://string-db.org/). Nodes represent proteins, and edges represent the evidence for the association, with line colors denoting different evidence sources: for example, magenta lines represent experimentally determined interactions, green lines represent gene neighborhood evidence.

To functionally characterize the PE-associated *BMAL1* gene, we analyzed the PPI network of its encoded protein, BMAL1 (also known as ARNTL), via the STRING database. The generated PPI network directly revealed BMAL1 as a central hub within the core circadian clock complex (Figure 1). Its key functional interactors—with a confidence score of 0.999 (the highest threshold in the database)—included CRY1, CLOCK, NPAS2, and CRY2 (Table 6). This finding confirms BMAL1’s central role in the core circadian transcriptional-translational feedback loop, which is mediated by its dimerization with CLOCK and negative regulation by the cryptochrome proteins CRY1 and CRY2.

## 3. Discussion

In this study, we identified a polymorphism within the *BMAL1* gene (rs11022780) that was significantly associated with the risk of PE. Further subgroup analysis revealed that this association was predominantly attributed to eoPE. Functional relevance of this variant was supported by cis-eQTL analysis, indicating its role in modulating *BMAL1* expression in whole blood. Additionally, PPI analysis further confirmed the central role of BMAL1 as a hub protein within the circadian rhythm network. In contrast, no significant associations were observed for the tested polymorphism in the *CLOCK* gene (rs1048004) or the other two *BMAL1* variants (rs4757144 and rs969485) with PE risk.

Our study identified a significant association between the *BMAL1* gene polymorphism (rs11022780) and PE, which was predominantly driven by the eoPE subtype. This finding lends genetic support to the growing evidence implicating circadian clock disruption, specifically through *BMAL1* dysregulation, in PE pathogenesis. The subtype-specificity of this association aligned with the distinct pathophysiological theories: the ‘placental’ theory for eoPE and the ‘maternal’ theory for loPE. The observed genetic risk likely contributed to the placental maldevelopment that characterizes eoPE through several interconnected mechanisms. As a core clock transcription factor, BMAL1 is known to influence cell proliferation and invasion. Given its documented role in modulating pathways like Wingless/Integrated (WNT)/β-catenin [60,61], a reduction in its expression may similarly disrupt key placental processes. This could manifest as impaired trophoblast invasion and spiral artery remodeling [17,62], which are central to the pathophysiology of eoPE. Furthermore, BMAL1 is crucial for maintaining redox homeostasis, and its deficiency is linked to attenuated antioxidant capacity and exacerbated oxidative damage [63,64]—a phenotype highly consistent with the severe placental ischemia–reperfusion injury and oxidative stress observed in eoPE [3,24,65]. Beyond placental and oxidative stress mechanisms, BMAL1 dysfunction may also impair vascular function by negatively influencing VEGF signaling [49] and promoting endothelial inflammation and dysfunction [51,66], thereby contributing to the systemic vascular abnormalities and hypertension in eoPE. In contrast, the lack of a significant association in the loPE subgroup reflected its more heterogeneous etiology, often linked to pre-existing maternal cardiometabolic dysfunction [32,33,34]. However, this interpretation had to be tempered by the results of our post hoc power analysis, which revealed limited statistical power for the loPE subgroup analyses (power = 25.4–29.3%). Consequently, the absence of a significant association for loPE should be viewed as inconclusive due to the high risk of a type II error, and a potential role for *BMAL1* in a subset of loPE cases cannot be ruled out. The significant association detected in eoPE, despite a borderline *p*-value and modest power (55.8–77.5%) for some genotype comparisons, suggests that the underlying genetic effect in this subtype is likely substantial enough to be detectable even in our cohort, yet it still warrants confirmation in larger, specifically powered studies. Collectively, our data suggest that *BMAL1* genetic variation may predispose to eoPE by disrupting core placental processes, including invasion, oxidative balance, and vascular function, while its role in loPE remains an open question necessitating future investigation in larger cohorts.

In line with a previous study of healthcare workers that identified the TT genotype of *BMAL1* rs11022775 as protective against hypertension (OR = 0.426) [57], our study found a significant associations of the *BMAL1* SNP rs11022780 with PE. Notably, under both codominant (TT vs. CC) and recessive (TT vs. CC + CT) genetic models, the TT genotype of rs11022780 conferred a protective effect against PE (aOR = 0.26 and 0.25, respectively). Although this SNP was located within an intronic region, further functional analyses indicated that the C allele of rs11022780 was correlated with decreased *BMAL1* mRNA expression in whole blood. Supporting the potential functional relevance of this variant, Burgermeister et al. reported that in metastatic colorectal cancer patients receiving bevacizumab (an anti-VEGF therapy), the TT genotype of rs11022780 was associated with longer overall survival, whereas the C allele predicted poorer clinical outcomes (HR = 1.61, *p* = 0.014) [67]. This finding gained additional biological plausibility given the known similarities between trophoblast behavior and tumor cell biology. As a core regulator of the molecular circadian clock, *BMAL1* was unique in that its single-gene ablation results in a complete loss of circadian rhythmicity [68]. In the present study, the C allele of rs11022780 was associated with reduced *BMAL1* expression in whole blood. The lower frequency of the protective genotype (TT for rs11022780) in PE cases suggests that reduced *BMAL1* expression may disrupt circadian rhythmicity and lead to downstream effects such as impaired VEGF signaling. These disturbances may represent a potential mechanism contributing to vascular dysfunction and inadequate placental remodeling, thereby possibly playing a role in the pathogenesis of PE [3,49,67]. While our findings suggest a protective mechanism mediated by *BMAL1* expression, the extrapolation of this eQTL evidence from whole blood to placental pathophysiology warrants careful consideration.

In this study, the eQTL evidence linking rs11022780 to *BMAL1* expression was derived from publicly available whole-blood datasets rather than placenta. We recognize that placental tissue is the most relevant for PE pathophysiology and that placenta-specific eQTLs would offer more direct evidence; indeed, dedicated placental eQTL studies have revealed numerous biologically significant eQTLs, some of which colocalize with loci for birthweight and other perinatal traits [69]. At the same time, large cross-tissue eQTL resources demonstrate that many cis-eQTLs are shared across diverse tissues, providing a practical justification for utilizing whole-blood data when placental eQTLs are unavailable [70]. Additionally, the use of whole-blood eQTLs offers a practical advantage, as blood sampling is non-invasive and more feasible for large-scale population studies compared with placental tissue collection. Moreover, core circadian genes including *BMAL1* and *CLOCK* are expressed in the human placenta and have established roles in placental function and PE pathogenesis [71], supporting the biological plausibility that genetic regulation of *BMAL1* may influence PE risk via placental mechanisms. While our genetic and eQTL data suggest that the rs11022780 locus influences PE risk by modulating *BMAL1* expression, future work is needed to deepen our understanding of this regulatory relationship through direct transcriptomic and proteomic analyses in disease-relevant models, including placental trophoblasts.

In contrast, our study did not reveal a significant association between PE susceptibility and the evaluated *CLOCK* SNPs—rs1048004. It is noteworthy that rs1048004 is located within the 3′-UTR region of the *CLOCK* gene, where sequence variations may influence mRNA stability or translational efficiency. Previous studies conducted in an American population have reported significant associations between this SNP and breast cancer risk [72]. This observation provides indirect evidence supporting the possibility that this loci possess regulatory functions. However, such effects appear not to extend to PE pathogenesis in our cohort. One potential explanation for this discrepancy lies in the phenotypic heterogeneity of PE. Supporting this view, Zhou et al. reported that *CLOCK* transcript levels were significantly reduced specifically in term PE placentas, but not in preterm cases, pointing to a potential subtype-specific association [36]. Our study population consisted predominantly of eoPE (n = 97) and loPE (n = 105) cases, with eoPE being particularly prone to result in preterm delivery [73]. Thus, the limited number of true term PE cases in our cohort may have attenuated the ability to detect an association with *CLOCK* gene polymorphism. Additionally, the absence of an association in the present study could be attributed to the possibility that the evaluated SNP may not capture the true functional variants within the *CLOCK* gene relevant to PE in the Chinese population. Genetic architecture, including allele frequencies and linkage disequilibrium patterns, often differs across ethnic groups. Therefore, risk loci identified in study of American populations may not be directly generalizable to Chinese individuals, owing to divergent genetic backgrounds and environmental influences.

Collectively, our results demonstrated that the rs11022780 locus in the *BMAL1* gene was significantly associated with PE, most notably with eoPE, and suggested that it may operate by modulating *BMAL1* expression, thereby providing novel molecular insights into the genetic architecture of the disease. Several limitations must be considered when interpreting these findings. As a hospital-based case–control investigation, the study may have been affected by admission bias. Moreover, although no significant association was detected between this SNP and late-onset PE, this absence of association likely reflects the limited sample size in the loPE subgroup rather than a true biological absence, underscoring the need for larger subtype-stratified analyses in the future. In addition, the eQTL analysis was based on whole-blood datasets rather than placental tissue, which is more directly relevant to PE; while cross-tissue studies have shown that many cis-eQTLs are shared across tissues, future validation using placenta-derived data will be important to confirm the observed regulatory relationship. The molecular consequences of this variant remain to be fully elucidated through direct transcriptomic or proteomic studies. Furthermore, data on peripartum management, such as antithrombotic therapy, were not collected, and their potential influence on outcomes was not assessed. Finally, our findings are derived from a single-center Chinese cohort; therefore, both independent validation in external populations and investigation in diverse ethnic groups are necessary to confirm the robustness and generalizability of the observed genetic effect.

## 4. Materials and Methods

### 4.1. Study Design and Participants

This hospital-based case–control study was conducted at two tertiary hospitals in Hunan Province, China, namely Hunan Provincial Maternal and Child Health Care Hospital and the Third Xiangya Hospital of Central South University. Between October 2020 and October 2023, a total of 202 women diagnosed with PE were enrolled as cases, along with 400 normotensive pregnant women recruited as controls from the same obstetric clinics. All participants strictly met predefined inclusion and exclusion criteria.

Preeclampsia was diagnosed by obstetricians based on the 2020 Chinese guidelines for hypertensive disorders in pregnancy [74]. According to the widely adopted clinical classification system, preeclampsia was further categorized into eoPE (diagnosed before 34 + 0 weeks of gestation) and loPE (diagnosed at or after 34 + 0 weeks of gestation) subtypes [75]. Participants were excluded if they had multifetal gestation, pre-existing chronic hypertension, or were unable to provide a blood sample or complete the questionnaire. Only those without kinship and who provided informed consent were included.

The study protocol received approval from the Ethics Committee of Xiangya School of Public Health, Central South University (Approval No. XYGW-2019-020), and was carried out in accordance with the principles of the Declaration of Helsinki. Written informed consent was obtained from every subject.

### 4.2. Data Collection

All investigators received standardized training before conducting the surveys. Data were collected through in-person interviews using a structured questionnaire, which covered maternal sociodemographic characteristics, reproductive history, lifestyle behaviors before and during pregnancy, autoimmune diseases and early-pregnancy infections. Operational definitions were provided for key variables of interest. For instance, pre-pregnancy BMI was categorized as underweight (<18.5 kg/m^2^), normal weight (18.5–24.0 kg/m^2^), or overweight/obesity (≥24.0 kg/m^2^) [76]. Smoking was defined as consuming at least one cigarette per week, while passive smoking referred to exposure to secondhand smoke for ≥10 min per week. Tea or alcohol consumption was classified as intake at least once per week or month, respectively. Sleep quality was rated as “good” (including very good, good, or fair) or “poor” (poor or very poor).

For genotyping, peripheral venous blood (3–5 mL) was collected from all individuals into ethylene diamine tetraacetic acid (EDTA)-anticoagulated tubes upon completion of the questionnaire. All samples were transported to the laboratory under refrigeration (4 °C) within 12 h. Subsequently, centrifugation was performed at 3500 rpm for 15 min to separate plasma and cellular components. Each fraction was aliquoted equally into three parts, assigned unique identifiers, and stored at −80 °C until further analysis.

### 4.3. SNPs Selection and Genotyping

The selection of SNPs in the *BMAL1* and *CLOCK* genes was based on the following criteria: (1) SNPs were required to have a minor allele frequency (MAF) ≥5% in East Asian populations, as identified from the dbSNP database on the NCBI website (https://www.ncbi.nlm.nih.gov/, accessed on 13 May 2022); (2) SNPs had to conform to HWE (*p* > 0.05) in our preliminary experiment; (3) SNPs must have functional relevance, supported by evidence from cis-eQTL data from the first phase of the eQTLGen consortium (https://eqtlgen.org/, accessed on 13 May 2022) [58] or prior literature documenting associations with disease. Based on these criteria, an initial set of seven SNPs was identified: four in *BMAL1* (rs4757144, rs11022780, rs969485, rs2290035) and three in *CLOCK* (rs1048004, rs10462028, rs7698022).

Genotyping of SNPs in the *BMAL1* and *CLOCK* genes was performed using the MassARRAY^®^ System (Agena Bioscience, San Diego, CA, USA), which utilizes matrix-assisted laser desorption/ionization time-of-flight mass spectrometry (MALDI-TOF MS) for detection. The primers for PCR amplification and single-base extension were designed using the Agena Assay Designer 4.0 software (Agena Bioscience, San Diego, CA, USA), based on the sequences flanking the target SNPs (primer sequences were provided in Supplementary Table S4). The experimental procedure followed the manufacturer’s standard protocol for the iPLEX^®^ Pro assay (Agena Bioscience, San Diego, CA, USA). Genomic DNA was amplified in a 5 μL multiplex PCR reaction, prepared by adding 1 μL of DNA (20 ng/μL) to 4 μL of Master Mix. The Master Mix contained 1.25 × PCR Buffer, 3.5 mM MgCl_2_, 500 μM dNTPs, 0.5 U HotStar Taq, and a primer mix at a concentration of 100 μM. Thermocycling was: 94 °C for 5 min; 45 cycles of 94 °C for 20 s, 56 °C for 30 s, 72 °C for 1 min; 72 °C for 3 min. Subsequently, 5 μL of PCR product was treated with 2 μL of SAP Mix (37 °C for 20 min; 85 °C for 5 min). A single-base extension was performed using the iPLEX^®^ Pro chemistry (Agena Bioscience, San Diego, CA, USA) by adding 2 μL of EXTEND Mix to the 7 μL SAP-treated product. The extension protocol was: 94 °C for 30 s; 40 cycles of 94 °C for 5 s, followed by 5 inner cycles of 52 °C for 5 s and 80 °C for 5 s; 72 °C for 3 min. The final extension products were purified, spotted onto a SpectroCHIP^®^ bioarray (Agena Bioscience, San Diego, CA, USA), and subjected to MALDI-TOF MS analysis on a MassARRAY Analyzer 4.0 (Agena Bioscience, San Diego, CA, USA). Genotype calls were automatically assigned by the TYPER^®^ 4.0 software (Agena Bioscience, San Diego, CA, USA) based on the mass differentials of the extension products.

Following genotyping, rigorous quality control was performed. This included testing for conformity to HWE (*p* > 0.05) and, critically, an assessment of LD. To ensure statistical independence and avoid collinearity, we performed LD-based pruning. When the pairwise LD (*r*^2^) between any two SNPs exceeded 0.8, only one tag SNP was retained from the correlated set. This process resulted in the exclusion of one SNP from *BMAL1* (rs2290035) and two from *CLOCK* (rs10462028, rs7698022).

Consequently, the final set for all downstream association analyses comprised three SNPs in *BMAL1* (rs4757144, rs11022780, rs969485) and one in *CLOCK* (rs1048004).

### 4.4. Statistical Analysis

To ensure data accuracy, all data were double-entered into the database using EpiData 3.0 (The EpiData Association, Odense, Denmark). Statistical analyses were carried out with IBM SPSS Statistics version 26.0 (IBM Corp., Armonk, NY, USA) and R version 4.5.0 (R Foundation for Statistical Computing, Vienna, Austria). Quantitative variables were reported as mean ± standard deviation, while categorical variables were summarized as frequency counts and percentages. Between-group differences for categorical variables were examined using chi-square tests. The HWE was tested for each SNP in the control group. Subsequently, to ensure variant independence and mitigate collinearity, pairwise LD was evaluated and used for pruning. This step retained one representative SNP from any set of variants with pairwise *r*^2^ > 0.8. Only loci conforming to both HWE (*p* > 0.05) and this LD criterion were included in the final analysis.

Associations between each SNP and PE were evaluated under codominant and recessive genetic models using both univariate and multivariate logistic regression, with results reported as OR and 95% CI. For genes harboring multiple independent SNPs after pruning, haplotype analysis was performed using Haploview v4.2 (Broad Institute, Cambridge, MA, USA) to investigate their combined effects on PE risk [77]. Furthermore, additive and multiplicative interactions between PE-associated SNPs and environmental factors (sleep quality during pregnancy and daily sleep duration during pregnancy) were evaluated using the interaction R package in R 4.5.0. Additive interaction was assessed using the relative excess risk due to interaction (RERI) and the attributable proportion due to interaction (AP). A significant additive interaction was indicated if the 95% CIs for RERI and AP excluded 0. Multiplicative interaction was evaluated through the inclusion of a product term in logistic regression models; a significant interaction was indicated by an OR with a 95% CI that did not include 1. All models for interaction analyses, both additive and multiplicative, were adjusted for potential confounders.

PE-associated statistically significant SNPs were subsequently analyzed in subgroups stratified by eoPE and loPE. EQTL analysis was performed using publicly available data from the first phase of the eQTLGen Consortium to investigate potential regulatory effects of PE-associated SNPs on *BMAL1* or *CLOCK* gene expression in whole blood [58]. Furthermore, PPI networks were constructed for proteins regulated by genes significantly associated with PE, using the STRING database (version 12, https://string-db.org/, accessed on 1 September 2025) [59].

## 5. Conclusions

This study identifies the *BMAL1* rs11022780 polymorphism to be associated with PE, with this association being notably observed in eoPE. Functional evidence indicates a potential role for this variant in mediating disease risk via regulatory effects on *BMAL1* expression. Protein interaction data further establish BMAL1 as a central component of the circadian rhythm network. Collectively, these results offer multilevel genetic and mechanistic insights into the role of *BMAL1* in PE pathogenesis, with particular relevance to eoPE cases. This work provides vital insights into PE’s etiology and holds promise for developing non-invasive genetic biomarkers for early risk stratification, which is crucial for improving pregnancy outcomes.

## Figures and Tables

**Table 1:** Baseline characteristics of pregnant women in the PE and control groups.

Characteristics	PE (n = 202)	Control (n = 400)	Statistical Value	*p* Value
Age (years)			33.891	<0.001 ***
<30	58 (28.71)	202 (50.50)		
30–35	88 (43.57)	148 (37.00)		
≥35	56 (27.72)	50 (12.50)		
Residence			24.120	<0.001 ***
Urban	170 (84.16)	383 (95.75)		
Rural	32 (15.84)	17 (4.25)		
Pre-pregnancy BMI (kg/m2)			83.977	<0.001 ***
Normal weight (18.5–24.0)	110 (54.46)	286 (71.50)		
Underweight (<18.5)	14 (6.93)	79 (19.75)		
Overweight/obesity (≥24.0)	78 (38.61)	35 (8.75)		
History of pregnancy complications			8.630	0.003 **
No	164 (81.19)	359 (89.75)		
Yes	38 (18.81)	41 (10.25)		
History of adverse pregnancy outcomes			2.891	0.089
No	105 (51.98)	237 (59.25)		
Yes	97 (48.02)	163 (40.75)		
History of anemia during pregnancy			4.284	0.038 *
No	164 (81.19)	350 (87.50)		
Yes	38 (18.81)	50 (12.50)		
Gravidity			1.921	0.166
1	73 (36.14)	168 (42.00)		
≥2	129 (63.86)	232 (58.00)		
Parity			0.007	0.932
0	123 (60.89)	245 (61.25)		
≥1	79 (39.11)	155 (38.75)		
Smoking during pregnancy			1.485	0.223
No	196 (97.03)	394 (98.50)		
Yes	6 (2.97)	6 (1.50)		
Secondhand smoke exposure during pregnancy			7.840	0.005 **
No	138 (68.32)	226 (56.50)		
Yes	64 (31.68)	174 (43.50)		
Alcohol consumption during pregnancy			2.956	0.086
No	196 (97.03)	375 (93.75)		
Yes	6 (2.97)	25 (6.25)		
Tea consumption during pregnancy			8.621	0.003 **
No	166 (82.18)	362 (90.50)		
Yes	36 (17.82)	38 (9.50)		
Folic acid supplementation during pregnancy			0.526	0.468
No	2 (0.99)	7 (1.75)		
Yes	200 (99.01)	393 (98.25)		
Sleep quality during pregnancy			28.341	<0.001 ***
Poor	62 (30.69)	51 (12.75)		
Good	140 (69.31)	349 (87.25)		
Daily sleep duration during pregnancy (hours/day)			32.588	<0.001 ***
<7	52 (25.74)	34 (8.50)		
≥7	150 (74.26)	366 (91.50)		
History of autoimmune disease			0.189	0.663
No	198 (98.02)	394 (98.50)		
Yes	4 (1.98)	6 (1.50)		
Fever in early pregnancy			1.503	0.220
No	190 (94.06)	385 (96.25)		
Yes	12 (5.94)	15 (3.75)		
Respiratory tract infection in early pregnancy			0.137	0.711
No	189 (93.56)	371 (92.75)		
Yes	13 (6.44)	29 (7.25)		
Gastrointestinal infection in early pregnancy			0.094	0.759
No	200 (99.01)	397 (99.25)		
Yes	2 (0.99)	3 (0.75)		
Periodontitis in early pregnancy			4.780	0.029 *
No	186 (92.08)	385 (96.25)		
Yes	16 (7.92)	15 (3.75)		
Urinary tract infection in early pregnancy			0.001	1.000
No	200 (99.01)	396 (99.00)		
Yes	2 (0.99)	4 (1.00)		
Reproductive tract infection in early pregnancy			7.975	0.005 **
No	180 (89.11)	381 (95.25)		
Yes	22 (10.89)	19 (4.75)		

Note: *: *p* < 0.05; **: *p* < 0.01; ***: *p* < 0.001. BMI: body mass index.

**Table 2:** Association between core circadian rhythm gene polymorphisms and PE risk under the codominant model.

Gene	SNP	Genotype	PE (n = 202)	Control (n = 400)	HWE *p* Value	OR (95% CI)	aOR (95% CI)
*BMAL1*	rs4757144	GG	80 (39.60)	148 (37.00)	0.647	1.00 (Ref.)	1.00 (Ref.)
		GA	94 (46.53)	187 (46.75)		0.93 (0.64, 1.34)	1.02 (0.66, 1.57)
		AA	28 (13.87)	65 (16.25)		0.80 (0.47, 1.34)	0.98 (0.54, 1.80)
*BMAL1*	rs11022780	CC	104 (51.49)	210 (52.50)	0.906	1.00 (Ref.)	1.00 (Ref.)
		CT	93 (46.04)	159 (39.75)		1.18 (0.83, 1.67)	1.09 (0.72, 1.64)
		TT	5 (2.47)	31 (7.75)		0.33 (0.12, 0.86) *	0.26 (0.09, 0.78) *
*BMAL1*	rs969485	GG	66 (32.67)	142 (35.50)	0.255	1.00 (Ref.)	1.00 (Ref.)
		GA	105 (51.98)	184 (46.00)		1.23 (0.84, 1.79)	1.21 (0.77, 1.89)
		AA	31 (15.35)	74 (18.50)		0.90 (0.54, 1.50)	0.65 (0.36, 1.19)
*CLOCK*	rs1048004	CC	173 (85.64)	352 (88.00)	0.250	1.00 (Ref.)	1.00 (Ref.)
		CA	28 (13.86)	45 (11.25)		1.27 (0.76, 2.10)	1.59 (0.88, 2.87)
		AA	1 (0.50)	3 (0.75)		0.68 (0.07, 6.57)	1.16 (0.09, 14.77)

Note: aOR was adjusted for the age, residence, pre-pregnancy BMI, history of pregnancy complications, history of anemia during pregnancy, secondhand smoke exposure during pregnancy, tea consumption during pregnancy, sleep quality during pregnancy, daily sleep duration during pregnancy, periodontitis in early pregnancy, reproductive tract infection in early pregnancy. *: *p* < 0.05. OR: odds ratio. CI: confidence interval.

**Table 3:** Association between core circadian rhythm gene polymorphisms and PE risk under the recessive model.

Gene	SNP	Genotype	PE (n = 202)	Control (n = 400)	OR (95% CI)	aOR (95% CI)
*BMAL1*	rs4757144	GG + GA	174 (86.14)	335 (83.75)	1.00 (Ref.)	1.00 (Ref.)
		AA	28 (13.86)	65 (16.25)	0.83 (0.51, 1.34)	0.97 (0.56, 1.70)
*BMAL1*	rs11022780	CC + CT	197 (97.52)	369 (92.25)	1.00 (Ref.)	1.00 (Ref.)
		TT	5 (2.48)	31 (7.75)	0.30 (0.12, 0.79) *	0.25 (0.09, 0.74) *
*BMAL1*	rs969485	GG + GA	171 (84.65)	326 (81.50)	1.00 (Ref.)	1.00 (Ref.)
		AA	31 (15.35)	74 (18.50)	0.80 (0.51, 1.26)	0.59 (0.34, 1.01)
*CLOCK*	rs1048004	CC + CA	201 (99.50)	397 (99.25)	1.00 (Ref.)	1.00 (Ref.)
		AA	1 (0.50)	3 (0.75)	0.66 (0.07, 6.37)	1.06 (0.08, 13.59)

Note: aOR was adjusted for the age, residence, pre-pregnancy BMI, history of pregnancy complications, history of anemia during pregnancy, secondhand smoke exposure during pregnancy, tea consumption during pregnancy, sleep quality during pregnancy, daily sleep duration during pregnancy, periodontitis in early pregnancy, reproductive tract infection in early pregnancy. *: *p* < 0.05.

**Table 4:** Subgroup analysis of the associations between the *BMAL1* rs11022780 polymorphism and PE risk by clinical onset time.

Gene & SNP	Genetic Model	Genotype	eoPE vs. Control		loPE vs. Control	
			aOR (95% CI)	*p* Value	aOR (95% CI)	*p* Value
*BMAL1* rs11022780	Codominant	CC	1.00 (Ref.)	0.029 *	1.00 (Ref.)	0.098
		CT	1.60 (0.95, 2.71)	0.078	0.70 (0.41, 1.21)	0.198
		TT	0.16 (0.02, 1.27)	0.083	0.28 (0.08, 1.01)	0.051
	Recessive	CC + CT	1.00 (Ref.)	0.048 *	1.00 (Ref.)	0.084
		TT	0.13 (0.02, 0.98) *		0.33 (0.09, 1.16)	

Note: aOR was adjusted for the age, residence, pre-pregnancy BMI, history of pregnancy complications, history of anemia during pregnancy, secondhand smoke exposure during pregnancy, tea consumption during pregnancy, sleep quality during pregnancy, daily sleep duration during pregnancy, periodontitis in early pregnancy, reproductive tract infection in early pregnancy. *: *p* < 0.05.

**Table 5:** The cis-eQTL analysis of PE-associated *BMAL1/ARNTL* rs11022780 polymorphism for gene expression in whole blood.

SNP	Samples	Assessed	Other	Z-Score	*p* Value	FDR
rs11022780	30,935	C	T	−5.269	1.372 × 10^−7^ ***	<0.001 ***

Note: ***: *p* < 0.001. FDR (false discovery rate) was used to adjust the *p*-values for multiple hypothesis testing. The cis-eQTL results presented in this table were sourced from the eQTLGen Consortium (Phase I) online platform (https://www.eqtlgen.org/) in accordance with its data use policy [58].

**Table 6:** PPI partners of BMAL1 (ARNTL) and their association scores from the STRING database.

Query Protein	Predicted Functional Partners	Coexpression	Experiments	Databases	Textmining	Homology	Score
BMAL1 (ARNTL)	CRY1	0.116	0.982	0.900	0.998	-	0.999
	NPAS2	0.110	0.919	0.800	0.991	0.640	0.999
	CLOCK	0.117	0.994	0.800	0.991	0.645	0.999
	CRY2	0.091	0.973	0.900	0.983	-	0.999
	SIRT1	0.049	0.345	0.750	0.987	-	0.997
	PER2	-	0.963	0.900	0.482	0.575	0.997
	CSNK1E	-	0.696	0.900	0.927	-	0.997
	NPAS4	-	0.585	0.900	0.795	0.560	0.990
	BHLHE41	-	0.089	0.500	0.978	-	0.989
	NR1D1	0.049	-	0.500	0.979	-	0.989

Note: Data in this table were obtained directly from the STRING database (version 12.0) [59] under a Creative Commons Attribution 4.0 International (CC BY 4.0 license).

## Data Availability

The data that support the findings of this study are available from the corresponding author upon reasonable request. The data are not publicly available due to privacy restrictions and confidentiality agreements.

## Supplementary Materials

The supporting information can be downloaded at: https://www.mdpi.com/article/10.3390/ijms262110797/s1, Table S1: Haplotype frequencies of *BMAL1* gene SNPs (rs4757144, rs11022780 and rs969485) and their associations with PE; Table S2: Additive and multiplicative interactions of the *BMAL1* rs11022780 polymorphism with sleep quality and sleep duration on PE risk; Table S3: Parameters and results of post-hoc power calculations for subgroup analyses of the *BMAL1* rs11022780 Polymorphism; Table S4: Primer sequences used for MassARRAY genotyping; Methods S1: Post-Hoc Power Analysis for Subgroup Analyses.
