# Supplementary material for: Association of BMAL1 and CLOCK Gene Polymorphisms with Preeclampsia Risk with Subtype Analysis
Source: Int J Mol Sci. 2025 Nov 6;26(21):10797. doi: 10.3390/ijms262110797 (PMC12610473; doi:10.3390/ijms262110797)
Supplement: Supplementary file 1 [file ijms-26-10797-s001.zip › ijms-3921151-supplementary.pdf]

Supplementary Table S1. Haplotype frequencies of *BMAL1* gene SNPs (rs4757144, rs11022780 and rs969485) and their associations with PE.

Haplotype	Overall frequency	Case frequency	Control frequency	χ^2	*p* Value
G–C–G	0.336	0.351	0.328	0.648	0.421
A–C–G	0.236	0.228	0.239	0.209	0.648
G–T–A	0.172	0.169	0.173	0.021	0.884
G–C–A	0.093	0.102	0.089	0.479	0.489
A–T–A	0.084	0.078	0.086	0.237	0.627
A–C–A	0.066	0.064	0.067	0.024	0.876
G–T–G	0.011	0.006	0.013	1.272	0.260

Note: Only haplotypes with a frequency >1% were shown. Haplotypes were constructed from SNPs rs4757144, rs11022780, and rs969485, in that order.

Supplementary Table S2. Additive and multiplicative interactions of the *BMAL1* rs11022780 polymorphism with sleep quality and sleep duration on PE risk.

Variable	*BMAL1* rs11022780		RERI [a]	AP [a]	Multiplicative Scale [a]
	CC+CT	TT			
Sleep quality during pregnancy			0.16 (-2.51, 0.66)	1.38 (-35.64, 22.67)	0.51 (0.05, 5.45)
Poor	1 (Ref.)	0.41 (0.06, 2.98)			
Good	0.55 (0.32, 0.94) *	0.11 (0.03, 0.45) **			
Daily sleep duration during pregnancy (hours/day)			0.07 (-3.64, 0.66)	1.03 (-77.7, 38.41)	0.30 (0.03, 3.30)
<7	1 (Ref.)	0.59 (0.08, 4.22)			
≥7	0.41 (0.22, 0.74) **	0.07 (0.02, 0.31) ***			

Note:[a] The RERI, AP and Multiplicative scale were adjusted for the age, residence, pre-pregnancy BMI, history of pregnancy complications, history of anemia during pregnancy, secondhand smoke exposure during pregnancy, tea consumption during pregnancy, periodontitis in early pregnancy, reproductive tract infection in early pregnancy. *: $p < 0.05$; **: $p < 0.01$; ***: $p < 0.001$.

Supplementary Methods S1: Post-Hoc Power Analysis for Subgroup Analyses

A post-hoc power analysis was conducted to evaluate the statistical power of the subgroup analyses for early-onset (eoPE) and late-onset (loPE) preeclampsia. The calculation was based on the standard formula for unmatched case-control studies, which incorporates the ratio of controls to cases (r). The formula for calculating the required number of *cases* is given by:

$$n = \frac{[Z_{1-\alpha/2}\sqrt{(1+\frac{1}{r})\bar{P}(1-\bar{P})} + Z_{\beta}\sqrt{P_1(1-P_1) + \frac{P_0(1-P_0)}{r}}]^2}{(P_1 - P_0)^2}$$

Where:

- n: Required sample size for the case group.
- r: Ratio of controls to cases ($n_{controls}/n_{cases}$).

- $Z_{1-\alpha/2}$: The critical value from the standard normal distribution for a two-sided Type I error rate (α) of 0.05, which is 1.96.
- Z_β: The critical value from the standard normal distribution corresponding to the desired statistical power (1 - β).
- P_0: The observed frequency of the exposure (i.e., the TT genotype) in the control group for the specific genetic model comparison.
- P_1: The observed frequency of the exposure in the case group (eoPE or loPE) for the specific genetic model comparison, obtained directly from study data.
- $\bar{P}$: The average exposure frequency, calculated as $\bar{P} = \frac{P_1 + rP_0}{1+r}$.

For this post-hoc analysis, the actual achieved sample sizes for cases (n) and controls (defining r), along with the observed genotype frequencies (P_0, P_1), were treated as known quantities. The above formula was used to solve for Z_β, which was then converted to the corresponding statistical power (1 - β) using the standard normal cumulative distribution function. Calculations were performed separately for different genetic models, as detailed in Supplementary Table S3.

Supplementary Table S3. Parameters and results of post-hoc power calculations for subgroup analyses of the *BMAL1* rs11022780 Polymorphism.

PE Subtype	Genetic Model	Comparison	Control Exposure (P_0)[1]	Case Exposure (P_1)[2]	Case Group (n)	Control: Case Ratio (r)	Statistical Power (1-β)
eoPE	Codominant	TT vs. CC	12.86%	2.27%	44	5.48	55.8%
eoPE	Recessive	TT vs. (CC+CT)	7.75%	1.03%	97	4.12	77.5%
loPE	Codominant	TT vs. CC	12.86%	6.15%	65	3.71	29.3%
loPE	Recessive	TT vs. (CC+CT)	7.75%	3.81%	105	3.81	25.4%

Note:[1] P_0 (Control Exposure): For the codominant model (TT vs. CC), the control group for comparison consists only of individuals with the CC or TT genotype, excluding CT heterozygotes. For the recessive model (TT vs. CC+CT), the control group includes all controls. [2] P_1 (Case Exposure): Calculated analogously to P_0, using case group numbers from the corresponding genetic model comparison.

Supplementary Table S4. Primer sequences used for MassARRAY genotyping.

SNP ID	Forward primer (5' to 3')	Reverse primer (5' to 3')
rs4757144	ACGTTGGATGTAGTGTTCTAGGCAGTTGGG	ACGTTGGATGGGTTTCTCTAGACTGTAGGC
rs11022780	ACGTTGGATGGTAAAATTGAGGCTCTAGGG	ACGTTGGATGTGCTGGTGGTCATGTTAGAG
rs969485	ACGTTGGATGGGAACTCTTGAATTGCTCCC	ACGTTGGATGATGTGAGTGGAAGAAGGGAG
rs2290035	ACGTTGGATGTTTTTCTCCCCTGGGCTTTC	ACGTTGGATGGGAGTTACAAAAAGAAAGGC
rs1048004	ACGTTGGATGGATAGTGTTAGGTTATCATC	ACGTTGGATGGACTGCTATCAGTCTCTTGG
rs10462028	ACGTTGGATGATGACAAAGGAGAGCACAGC	ACGTTGGATGATACTACGGTCTAGCCTTCG
rs7698022	ACGTTGGATGGAGGCTATAAGAGTAAGTT	ACGTTGGATGGTGTAACTTCTGTGAAACAGG
